# A toolkit for capturing a representative and equitable sample in health research

**DOI:** 10.1038/s41591-023-02665-1

**Published:** 2023-12-08

**Authors:** Ameeta Retzer, Bircan Ciytak, Foram Khatsuria, Juma El-awaisi, Isobel M. Harris, Laura Chapman, Tony Kelly, Jenny Richards, Emily Lam, Philip N. Newsome, Melanie Calvert, NIHR Birmingham Biomedical Research Centre REP-EQUITY Group, Juma El-awaisi, Andrew Filer, Shishir Shetty, Jo Parish, Steve Watson, Elizabeth Sapey, Caroline Gillet, Jo Palmer, Zehra Yonel, Zohur Miah, Joseph Alderman, Elinor Laws, Xiaoxuan Liu

**Affiliations:** 1 Institute of Applied Health Research, University of Birmingham, Birmingham, UK; 2 Centre for Patient Reported Outcomes Research, Institute of Applied Health Research, University of Birmingham, Birmingham, UK; 3 National Institute for Health and Care Research (NIHR) Applied Research Collaboration West Midlands, Birmingham, UK; 4 NIHR Birmingham Biomedical Research Centre (BRC), University of Birmingham, Birmingham, UK; 5 Institute of Cardiovascular Sciences, University of Birmingham, Birmingham, UK; 6 Centre for Liver and Gastrointestinal Research, Institute of Immunology and Immunotherapy, University of Birmingham, Birmingham, UK; 7 Birmingham Health Partners Centre for Regulatory Science and Innovation, University of Birmingham, Birmingham, UK; 8 Midlands Health Data Research UK, Birmingham, UK; 9 NIHR Blood and Transplant Research Unit (BTRU) in Precision Transplant and Cellular Therapeutics, University of Birmingham, Birmingham, UK; 10 Rheumatology Research Group, Institute of Inflammation and Ageing, University of Birmingham, Birmingham, UK; 11 Institute of Cancer and Genomic Sciences, University of Birmingham, Birmingham, UK; 12 British Heart Foundation, Birmingham, UK; 13 Institute of Inflammation and Ageing, University of Birmingham, Birmingham, UK; 14 Institute of Metabolism and Systems Research, University of Birmingham, Birmingham, UK; 15 University Hospitals Birmingham National Health Service (NHS) Foundation Trust, Birmingham, UK; 16 Institute of Clinical Sciences, School of Dentistry, University of Birmingham, Birmingham, UK

**Keywords:** Clinical trial design, Scientific community, Research data

## Abstract

Research participants often do not represent the general population. Systematic exclusion of particular groups from research limits the generalizability of research findings and perpetuates health inequalities. Groups considered underserved by research include those whose inclusion is lower than expected based on population estimates, those with a high healthcare burden but limited research participation opportunities and those whose healthcare engagement is less than others. The REP-EQUITY toolkit guides representative and equitable inclusion in research. The toolkit was developed through a methodological systematic review and synthesis and finalized in a consensus workshop with 24 participants. The REP-EQUITY toolkit describes seven steps for investigators to consider in facilitating representative and equitable sample selection. This includes clearly defining (1) the relevant underserved groups, (2) the aims relating to equity and representativeness, (3) the sample proportion of individuals with characteristics associated with being underserved by research, (4) the recruitment goals, (5) the strategies by which external factors will be managed, (6) the methods by which representation in the final sample will be evaluated and (7) the legacy of having used the toolkit. Using the REP-EQUITY toolkit could promote trust between communities and research institutions, increase diverse participation in research and improve the generalizability of health research. National Institute for Health and Care Research PROSPERO identifier: CRD42022355391.

## Abstract (web summary)

Developed through systematic review and expert consensus, the REP-EQUITY toolkit provides a seven-step guide for investigators to facilitate representative and equitable recruitment into clinical research studies.

## Main

Several characteristics could contribute to individuals and groups being underserved by research in context- and study-specific circumstances. These include demographic (for example, age, ethnicity, gender identity), social and economic (for example, employment status, living location, educational attainment), disease-specific (for example, having rare diseases) and health status-related (for example, having mental health conditions, having multimorbidities, being pregnant) characteristics^1^. Failure to include research participants who represent target populations limits the generalizability of research findings^2^. Different groups might respond differently to an intervention for a range of reasons. Without representative participation, clinical trials cannot capture differences in interventional response^1^. This can introduce bias and affect research quality, resulting in a reluctance to offer interventions to specific groups^3^ and contributing to health inequality and inequity^4^.

Although improving the diversity of clinical trial participants is increasingly prioritized, the coronavirus disease (COVID-19) pandemic accelerated the need to generalize research findings to groups experiencing the greatest health burdens^5^. Minoritized populations, such as Black African and Caribbean and Southeast Asian groups in the UK^6^, as well as Black, Hispanic and Native American communities in the United States^7^, experienced disproportionate risks of severe COVID-19 complications and death yet are generally underrepresented in COVID-19 research^8^. Underrepresentation is endemic in health research (for example, for groups marginalized by ethnic and racial status^9^, gender^10,11^, age^12^ and having comorbidities^13^ or severe mental illness^14^), limiting the value of research evidence when applied in broader clinical contexts.

Initiatives to improve research representativeness have emerged through an increased understanding of structural inequities resulting from historical and ongoing discriminatory practices^15^ and their continued impact on individual and group outcomes. Equity focus in a healthcare context means deliberately considering the impact of research design and implementation, policies and practice on underserved groups to identify and address complex and multifactorial barriers^16^. The US National Institutes of Health Revitalization Act of 1993 acknowledged the underrepresentation of women and minority groups and addressed clinical research equity by requiring justification for their exclusion^17^. Subsequently, the US Food and Drug Administration encouraged the inclusion of underserved groups, including older adults^18^, women^19^ and minority racial and ethnic groups^20^, through interrogating inclusion criteria, promoting accessibility^21^ and adopting an equity focus in specific clinical areas^22^. In the UK, efforts have been led by the National Institute for Health and Care Research (NIHR), including through guidance development^23,24^.

**Fig. 1:**
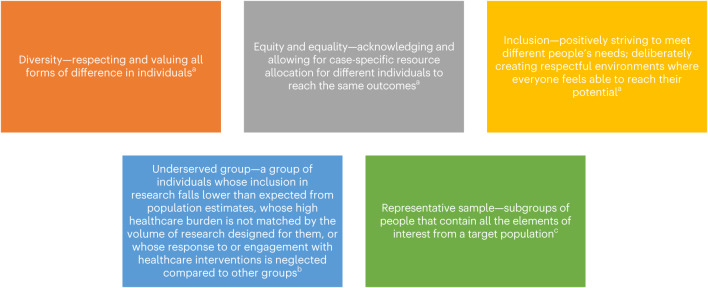
Key definitions. Definitions were adapted from ^a^Calvert et al.^68^, ^b^Morris et al.^43^ and ^c^Enticott et al.^69^.

However, with growing emphasis placed on representative inclusion in research by the public and research funders, a practical, evidence-based approach to capture inclusive and representative research samples, minimize bias and promote equity is required^3^. This would avoid a mechanistic approach that neglects generalizability and further undermines informed consent^25^ and trust between underserved groups and research institutions^26^. This study aimed to develop such guidance through a systematic review of existing methodology, synthesis of the REP-EQUITY toolkit, and a consensus workshop to finalize the toolkit and formulate implementation considerations. The terms used are defined in Fig. 1.

## Results

### Patient and public involvement

Four patients and public members of diverse backgrounds ensured that project aims, conduct and outputs reflected their interests. Based on their advice, the toolkit was developed centered on the research inclusion of underserved groups, focusing on equity rather than equality.

### Systematic review

MEDLINE and Embase were searched until September 2022 and October 2022, respectively, yielding 2,209 studies. Reference and citation searching yielded four studies. Gray literature databases were searched until August 2022 (Google Search, Google Images and Google Scholar) and September 2022 (Trip database, National Grey Literature Collection database, BASE database), which yielded one document. A total of 356 duplicates were removed. Fifty-four full-text articles were retrieved for screening. Seven academic papers^27–33^ and one gray literature document^34^ were included in the review (Supplementary Fig. 1).

The characteristics of the included documents are summarized in Supplementary Table 1. One document was developed in Australia^27^, one in the Republic of Ireland^28^, two in the UK^29,34^ and four in the United States^30–33^. Of those developed in the United States, two were formulated in the context of the US National Institutes of Health guidelines on the inclusion of minority groups in research^30,31^. Five documents are related to including individuals from minority ethnic and racial groups^28,29,31,32,34^, and three were developed with a specific interest in transgender and nonbinary individuals^32^, women^31^, and undocumented migrants, refugees, people seeking protection (asylum seekers) and low-income economic migrants^28^. One paper is related to older adults^33^, one to underrepresented groups^30^, and one to groups considered marginalized or vulnerable in high-income and lower- and middle-income countries^27^. Two papers are related to socioeconomic status and rural populations^30,33^. Two papers are related to cancer clinical trials^29,30^, one to health and social care research^34^, one to clinical research^31^, one to human immunodeficiency virus (HIV)^32^, one to global health research^27^, one to primary healthcare research^28^ and one to Alzheimer’s disease^33^.

Four documents describe frameworks^27,30,31,34^, and four describe methods used to capture a representative sample inclusive of underserved groups^28,29,32,33^. Of the four frameworks, three have an equity focus, two of which guide how to establish scope and aims in relation to equity^30,31^ and one guides how to formulate an equitable proposal^27^. Of the four papers describing methods, one is related to the design and implementation of a participant recruitment registry for clinical studies^33^, two describe participant enrichment strategies in trial settings^28,32^ and one describes recruitment into trials^29^. Four documents used a literature review design, three to formulate a framework^27,30,34^ and one to inform trial design^32^. All documents refer to using prevalence and population data, with the four frameworks advising on using such data to inform proposal development. Of the articles reporting methods, three used these data to establish criteria in advance and one to verify representativeness^29^. Two articles describe ascertaining target groups using prevalence data or particular study aims and recruiting patient and public advisors representative of these^28,32^. The authors of one paper convened a stakeholder panel that included patients and the public^32^.

Quality appraisal and risk-of-bias assessment were planned but not possible due to the unavailability of relevant appraisal tools.

### Consensus workshop

The patient and public panel advised on the workshop format and venue. A preparation session was attended by three public contributors and the public involvement manager. The 2-h hybrid-format workshop took place on November 14, 2022. The 24 participants included 4 public contributors, 1 public involvement manager, 17 researchers from the Birmingham Biomedical Research Centre (BRC) research areas (inflammatory arthritis; sarcopenia and multimorbidity; inflammatory liver disease; cancer inflammation; patient-reported outcomes; data, diagnostics and decision tools; infection and acute care; metabolic health in women; thrombo-inflammation; oral, intestinal and systemic health; and next-generation therapies) and 2 operational team members. Twelve participants convened in person at the University of Birmingham, and 12 joined through videoconferencing software.

### REP-EQUITY toolkit

The seven-part draft REP-EQUITY toolkit reflected the research design pathway, identifying (1) relevant groups, (2) aims, (3) sample requirements, (4) recruitment goal considerations, (5) external factor management, (6) evaluation and (7) legacy. Workshop participants unanimously agreed on the toolkit’s content and utility in informing the selection of representative research samples.

**Fig. 2:**
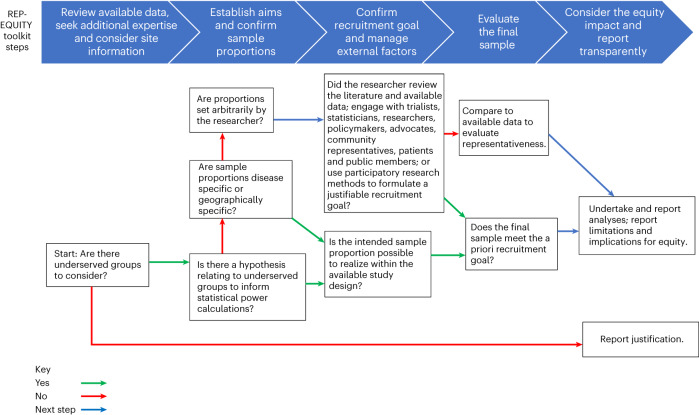
Algorithmic description of the REP-EQUITY toolkit. The process to follow based on the toolkit is presented as questions and pathways posed to the researcher. These ascertain researcher intentions and navigate to options to enable representative and equitable sample selection, indicated by differently colored arrows.

An algorithmic description of the REP-EQUITY toolkit is shown in Fig. 2. A checklist for its use by research teams during protocol development and final trial reporting is presented in Table 1. Users can use the checklist to record how each consideration has been addressed or justify where this was not possible. The REP-EQUITY toolkit consists of seven questions that may be considered sequentially, although elements will interact and influence decisions at earlier and later stages. The questions, options and considerations guide the user through the process of formulating a strategy to capture a representative and equitable sample in a particular research context and consider potential limitations during study design, facilitating transparent reporting. The process was iterated and is exemplified through the development of a retrospective case-study (Table 2).

**Table 1:** REP-EQUITY toolkit checklist

Section	REP-EQUITY question	Explanation	How this has been considered and addressed by the research team
Participant and site sampling	1. What are the relevant underserved groups?	Identify relevant groups and/or characteristics using available data^33,34^ and additional expertise^28,32^, depending on sites of interest or availability of sites^27,32^. Use this to inform and justify the choice of personal data to be collected from participants.	
Objectives	2. What is the aim in relation to representativeness and equity?	Define the aim in terms of whether it is to test hypotheses about possible differences by underserved characteristic(s), generate hypotheses about possible differences by underserved characteristic(s), or ensure a just and equitable distribution of the risks and benefits of participation in research^31^.	
Participant and site sampling	3. How will the sample proportion of individuals with underserved characteristics be defined?	Justify the chosen proportion in terms of comparability across studies, generalizability to population(s) of interest, distribution and equity impact of the risks and benefits of research participation, and feasibility of approach^30^.	
Participant and site sampling	4. What are the recruitment goals?	Define recruitment goals in terms of requirements for statistical power calculations, exploratory analyses, generalizability^30,31^ and how they might be practically and ethically realized^30^.	
Participant and site sampling	5. How will external factors be managed?	Formulate strategies to mediate external factors that may influence whether efforts to capture an equitable and representative sample can be realized^30^.	
Analyses	6. How will representation be evaluated?	State the means for evaluation (for example, match with a priori recruitment goal^30,31^ or available data, compare to available data to establish the impact on equity in terms of burdens and benefits of research participation^30,31^).	
Impact and dissemination	7. What will be the legacy?	Plan for lasting outputs (for example, advisory groups^32^, participant registries^33^, relationships with communities^28,32^); transparently report the use of the toolkit and data and/or findings relating to underserved groups.

**Table 2:** REP-EQUITY worked case study example

REP-EQUITY question	Retrospective response
1. What are the relevant underserved groups?	A literature review identified a range of potentially underserved groups, including individuals with cirrhosis and associated conditions^55–58^, specific racial and/or ethnic groups (Hispanic/Latinx, white^59^, Japanese^60^, Bangladeshi^61^), groups by gender (men, varying risk in women^62^) and sexual minority groups (men who have sex with men and transgender women (relating to hepatitis risk)^63,64^). A team in Birmingham, UK, led the trial; trial participants were recruited from the UK population. Trial initiation predated currently available epidemiological datasets such as CPRD^65^ and novel specialized software such as DExtER (data extraction for epidemiological research)^66^, but information identified from the literature review is available and could now be used in combination with these resources.
2. What is the aim in relation to representativeness and equity?	No specific hypotheses relating to underserved groups were made; thus, the aim could have been to attain a just and equitable distribution of the risks and benefits of research or to undertake exploratory analyses.
3. How will the sample proportion of individuals with underserved characteristics be defined?	The trial was funded by the NIHR and a UK charity; thus, proportions may reasonably be derived from UK populations, adjusted for disease prevalence. The most common causes of liver cirrhosis are alcohol consumption, diet and hepatitis; thus, proportions could be established based on the resulting health burden.
4. What are the recruitment goals?	The trial sample size (*n* = 81) was derived from power calculations to detect clinically important effects on liver function. The trial eligibility criteria were comprehensive and included alcohol-related liver disease (ARLD), hepatitis C, hepatitis B, primary biliary cholangitis, hemochromatosis, cryptogenic cirrhosis, nonalcoholic fatty liver disease and/or α_1_-antitrypsin deficiency as the liver disease etiology. Stratification was not used. No particular recruitment goals were set except for recruitment to reflect health burden by etiology, which would influence recruitment goals related to underserved groups. Etiology interacts with underserved characteristics in the population (for example, hepatitis and alcohol consumption). If stratification was used, it may be reasonable to formulate recruitment goals relating to alcohol consumption, diet and hepatitis.
5. How will external factors be managed?	Stem cell research is limited by the availability of facilities required for storage; thus, sites were selected on this basis and under preexisting collaborative arrangements. Depending on the intended etiological proportions, the selected sites (Birmingham, Nottingham, Edinburgh (UK)) and their expertise relating to specific groups may be assessed for the extent to which recruitment goals are viable. Exclusion criteria relating to hepatitis C infection and antiviral time present barriers to trial entry for certain groups.
6. How will representation be evaluated?	The final sample included 53 men and 28 women. Age was reported, whereas ethnicity was not reported. The causes of liver disease were ARLD (*n* = 38), hepatitis C (*n* = 10) and others (*n* = 33). The final sample could have been evaluated against available data to evaluate representativeness in the context of trial aims and monitored during the trial to allow adjustments as required. The proportion of those with ARLD may be representative of the disease burden.
7. What will be the legacy?	Rationale and methods to formulate sample proportions were not reported, but the sample characteristics reported allow for appraisal of the applicability of results and meta-analyses. Retrospective use of tools for equality impact assessment will enable consideration of equity implications.

Case study—“Granulocyte colony-stimulating factor and autologous CD133-positive stem-cell therapy in liver cirrhosis (REALISTIC): an open-label, randomised, controlled phase 2 trial” (ref. ^67^).

#### What are the relevant underserved groups?

An understanding of which underserved groups are relevant to a research topic can be established through reviewing available data (for example, prevalence estimates, surveillance data, primary and secondary care data, academic evidence base and gray literature)^33,34^, seeking additional expertise (for example, by engaging with advocates, scientists, community representatives, policymakers, patients and public members^28,32^, and through participatory research) and considering available research sites and sites of interest (in terms of prevalence of disease, experience of site personnel^32^ and promotion of equity depending on the differential health burden^27^). Use of these data will interact with the clarification of aims, the definition of the proportion of individuals with underserved characteristics (including, but not limited to, social and structural factors (for example, sociopolitical, economic and historical)), the considerations for recruitment goals and the evaluation of the final sample. For equitable research site partnership, the gap in health and well-being between the lead institution and potential sites and the generalizability of research findings should be considered^27^. This will inform and justify the choice of personal data to be collected from participants to monitor and report sample characteristics.

#### What is the aim in relation to representativeness and equity?

Researchers should decide whether the aim in relation to representativeness and equity is to test hypotheses about possible differences by underserved characteristic(s), generate hypotheses about possible differences by underserved characteristic(s), or ensure a just and equitable distribution of the risks and benefits of participation in research^31^. Depending on the aim, researchers may either ensure adequate statistical power to test hypotheses on differences, conduct exploratory analyses to generate hypotheses, or select study participants such that results can be generalized to relevant populations^31^.

#### How will the sample proportion of individuals with underserved characteristics be defined?

The sample proportions can be defined as decided by the researchers, based on populations with a specific disease or clinical indication, based on the geographic proportion of underserved groups (nationally, locally or institutionally), or by adjusting for disease prevalence or mortality, where required, using published literature or health data sources^30^. In doing so, researchers should consider choosing the sample proportion in terms of comparability across studies, generalizability to populations of interest, distribution of the risks and benefits of research participation and how this affects equity, and the feasibility of the approach^30^.

#### What are the recruitment goals?

Recruitment goals will depend on whether they are defined by statistical power calculations, exploratory analyses or generalizability considerations^30,31^. Upon confirmation of the recruitment goals, researchers should consider how their fulfillment interacts with the communities’ disposition toward research, the potential for further alienation and the conduct of upholding informed consent^30^. Cases in which recruitment goals are not formulated through sample size calculations require consideration of how the intended sample proportions will be managed within the available study design. The approach may be determined by the nature of the study (for example, experimental medicine versus later-phase trials). Smaller studies may require a matrix approach that improves representation. This may be developed pragmatically in collaboration with trialists, advocates, researchers, community representatives, policymakers, patients and public members.

#### How will external factors be managed?

External factors are those that might affect the ability to recruit an equitable and representative sample. These factors include, but are not limited to, underreporting or availability of data pertaining to indicators of inequality and disadvantage, research study timeline, independent review boards, participant retention, disease-specific factors (for example, the requirement for representation of different clinical characteristics or etiology)^29,30^ and preexisting institutional collaboration agreements that may limit site selection. Once external factors are identified, corresponding strategies to counter these barriers should be generated.

#### How will representation be evaluated?

The final sample’s representativeness can be evaluated by matching with a priori recruitment goals^30,31^, verifying against available population-level demographic data adjusted for disease prevalence^29^ and comparing to available data to establish the impact on equity in terms of the burdens and benefits of research participation^30,31^. Transparent reporting of these methods allows for assessing across-study comparability, generalizability and equity^30^.

#### What will be the legacy?

Efforts to capture a representative and equitable sample can have lasting outputs that further promote good practice and inform and direct future efforts. These include the formation of advisory groups^32^, participant registries^33^ and relationships with communities^28,32^ during the research course, as well as the contribution to the evidence base upon completion, which will inform and direct future efforts and further research.

**Fig. 3:**
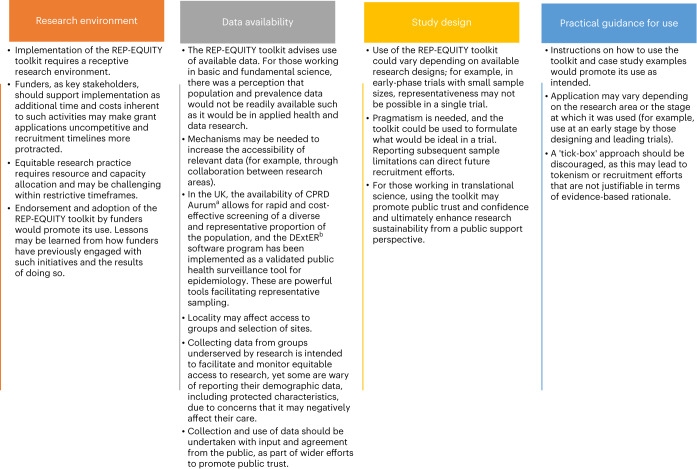
Implementation considerations. ^a^CPRD Aurum^70^, ^b^DExtER^66^.

Workshop participants highlighted the barriers and facilitators to using the REP-EQUITY toolkit, relating to the research environment, data availability, research area and study design, and availability of practical guidance for use, all of which have implications for the eventual utility and implementation of the toolkit (Fig. 3).

## Discussion

The REP-EQUITY toolkit provides an evidence-based approach to recruiting a representative and equitable research cohort, including those underserved. It was developed through the identification and synthesis of eight methodological documents and finalized during a consensus workshop. The REP-EQUITY toolkit consists of seven distinct steps forming stages of the research design pathway, facilitating consideration of (1) relevant groups, (2) aims, (3) sample requirements, (4) recruitment goals, (5) external factors, (6) evaluation and (7) legacy.

Presented as a checklist, the REP-EQUITY toolkit enables investigators to record how the research team has addressed each consideration or justify where this was not possible. The REP-EQUITY toolkit is intended to guide the user through a reflective process of formulating a strategy and the potential limitations during study design and conduct. This facilitates transparency, promotes trust and directs future efforts for representative and equitable research. This complements the substantial existing literature on engaging, retaining^35–37^ and conceptualizing underserved groups^38,39^ and assessing equity^23^. Unless these are operationalized alongside the REP-EQUITY toolkit, equity and representativeness in research cannot be achieved. Resources such as the Clinical Practice Research Datalink (CPRD) Aurum database are increasingly used to facilitate the enrollment of research participants representative of the real-world population through phenotype specification, systematic code searching, iterative and clinical review, and analysis of prevalence and validation^40^. Technological advances of this kind, used in combination with and informed by literature review and engagement with experts (including those with lived experience to identify underserved groups in particular contexts), maximize the potential utility and impact of the REP-EQUITY toolkit. Using a range of complementary resources to determine sample characteristics and proportions permits a comprehensive, transparent and inclusive approach, with each resource offsetting any potential shortcomings of the others. For example, resources such as CPRD Aurum are not informative of the wide range of characteristics that may result in individuals being underserved. Meanwhile, published literature and topic experts may not be able to provide easily quantifiable information. Although there are practical difficulties when aiming for representative samples (due to the challenge of defining groups and nomenclature, the intersectionality of identity, the context-specific nature of being underserved and the issue of missing data), using the REP-EQUITY toolkit will allow the generation of case studies describing how these can be managed.

This study has some methodological strengths and limitations that should be considered. Pertinent articles may have been missed in the literature search. Although this was mitigated through backward and forward citation searching, extensive searching of gray literature sources and developing strategies from previous systematic reviews, non-English-language literature was excluded. In addition, although several gray literature databases were used, searches through Google would be geotagged and linked with UK sources. Therefore, the documents included in this study may be unrepresentative of the international nonacademic literature. The research undertaken in Birmingham BRC is representative of a broad range of research areas affecting a diverse range of individuals and patient groups, and all researchers have experience in research relating to populations residing in Birmingham, UK, a superdiverse city^41^. However, as workshop participants were recruited from a high-resource research center, their recruitment only related to their involvement in the NIHR Birmingham BRC research themes rather than their personal and sociocultural characteristics or the experience of low- and middle-income countries and resource-limited settings (these data were also not collected). The degree of bias presented by this is unknown. Therefore, future workshops with international stakeholders representing these perspectives and interests are recommended as the next step to enhance the output and expand the REP-EQUITY toolkit to global applicability. Quality appraisal was not possible due to the unavailability of relevant appraisal tools, although the methodological approaches of the documents included in the systematic review were extracted and reported. The limited guidance available for methodological systematic reviews is an ongoing challenge^42^.

Assessing the acceptability and usability of a methodological guidance can allow further iteration to promote its implementation^43^. Standards for methodological guidance development advise seeking feedback and criticism after publication^44^, an important step to understanding the usability and value of a tool^43^. Exploring implementation during toolkit development, such as in this research, is novel. Using a consensus workshop to explore the facilitators and barriers to implementing the toolkit permitted reflection on how its use may be promoted. Although these findings were drawn from participants from a biomedical research setting, the use of the toolkit should be considered throughout health research. Workshop participants discussed differences in how the REP-EQUITY toolkit may be used in applied health research and health data science. Documenting the use of the REP-EQUITY toolkit in other fields of health research, such as applied health research and global health, will facilitate the generation of practical case studies and the continued iteration of the toolkit. Future iterations could include sources of contemporary discourse in equity-centered research, such as reflexive exercises on the positionality of the research teams^45^ using the toolkit and exploring the addition of adherence levels to the items. We aim for the toolkit to evolve as related standards become established. Further validation and piloting of the REP-EQUITY toolkit and tracking of its use will further maximize its potential utility. The final step of the toolkit referring to legacy is particularly important.

In studies with small sample sizes, such as in experimental medicine, the extent to which the sample can meaningfully represent a population may be limited. In these cases, the toolkit can support the formulation of aims and the transparent reporting of evaluation, which will be crucial to directing future efforts to enable the generation of representative research outputs. This represents a shift in research practice in which the burden for one study to be representative of all is considerably minimized. Consideration of whether a single study may capture a representative sample that includes underserved groups, paired with ongoing interrogation of who remains underserved by research and who is yet to be considered, will inform continued use of the toolkit.

With a greater understanding of how research practices interact with health outcomes and the perpetuation of health inequality and inequity, the REP-EQUITY toolkit facilitates an evidence-based approach that integrates lived experience to formulate a rationale for the inclusion of underserved groups. Many groups remain skeptical and wary of research, particularly relating to the collection of their personal, socioeconomic and cultural data that regularly form the basis for discriminatory practice^46^ and are associated with poor health outcomes^47^. Using the REP-EQUITY toolkit may build trust with those underserved and wary of participation in research and the use of research outputs, as well as direct efforts to groups for which there will be the greatest benefit in specific health areas from an equity standpoint. Examples of this include the COVID-19 vaccination hesitancy experienced by minority ethnic and racial groups^48^, particularly intersecting with health status (for example, HIV infection^49^ or pregnancy^50^) and in prior cases such as polio vaccination internationally^51^, which unevenly distributed the benefits of vaccination research. For some, legacies of historical atrocities, such as the Tuskegee Syphilis Study, and ongoing discrimination contribute to continued mistrust of health and research institutions, adversely affecting participation^52^.

The workshop identified a range of factors (relating to the research environment, data availability, research area and study design, and practice guidance development) that might serve as barriers and levers to the use of the toolkit. Formal adoption of the REP-EQUITY toolkit by the research community requires support from funders as its use affects resources in terms of finances, time, capacity and effort; as attaining equity requires this support for different individuals to reach the same outcomes^53^. This might mean making decisions about delivering fewer but higher-quality studies. However, without methodological intervention, such as through the use of this toolkit, research samples will remain biased due to limited generalizability, further contributing to research waste^54^ and continued exacerbation of health disparity and burden. Use can be further promoted through the adoption and championing of the REP-EQUITY toolkit by policymakers, national funding bodies, ethics committees and public contributors to research. Furthermore, integration with other existing mechanisms to uphold methodological rigor could be explored.

## Methods

The REP-EQUITY toolkit was formulated and finalized in two stages: a comprehensive literature search informed by systematic review methodology^63^ and a consensus workshop involving researchers and theme leads from the NIHR Birmingham BRC and members of the patient and public involvement and engagement (PPIE) panel. Methods are reported in accordance with accepted guidelines^70–72^.

### Ethical approval

This study has undergone ethical review by the Research Ethics Committee at the University of Birmingham and was granted full approval in October 2022 (ERN_22-1182).

### PPIE

The NIHR Birmingham BRC convened an equity, diversity and inclusion PPIE panel for the purposes of this study. Panel members had personal and professional links with communities that are underserved by research. Although personal data were collected from panel members, including age, self-reported gender and ethnicity, educational attainment, and previous research involvement and participation, their selection for the panel was not based on these characteristics. During the study, the panel met with the research team monthly in dedicated meetings. They advised on conducting and delivering the research, refining the project scope and aims, selecting the gray literature search terms, including public participants in the consensus workshop and formulating key messages for dissemination. Panel members were invited to join the consensus workshop as participants.

### Systematic review search strategies

Our aim was to identify methodological frameworks and approaches used to guide the capture of a representative participant sample in health research, inclusive of groups underserved by research.

Embase and MEDLINE were searched, without date limitation, to identify academic literature. The search strategy included MeSH (Medical Subject Headings) terms, broad search terms, and phrases and keywords related to equity, diversity and inclusion in health research, accounting for variation in terminology used in this area (Supplementary Tables 2 and 3). These were refined through scoping searches, informed by strategies used in comparable systematic reviews^73,74^. Forward and backward citation searching was used. Google (Google Search, Google Images and Google Scholar), Trip database^75^, National Grey Literature Collection database^76^ and BASE database^77^ were used to search gray literature. Search terms and keywords were derived from the academic literature review search strategy and refined iteratively in consultation with search specialists and the PPIE panel. Search terms were piloted in each database in a process in which the retrieved records were checked for sensitivity, terms were reworded and reentered, and items related to our aim were retrieved (Supplementary Table 4). Upon advice from the PPIE panel, new terms were introduced based on those commonly used in the UK relating to ethnicity, reflecting categories used in census data.

### Document selection and eligibility criteria

Academic literature records were downloaded into Endnote (version X9), and duplicates were removed. Titles, abstracts and full texts were screened by two independent investigators (B.C., F.K.). The first 100 results from searches of gray literature databases were screened independently by a researcher, and a 10% sample of these was screened by a second researcher using a minimum agreement threshold of 90%. Disagreements on the eligibility of academic and gray literature documents were resolved through discussion, including a third reviewer (A.R.) when required.

Papers were eligible if they were published in the English language, included guidelines and/or toolkits, or reported methods for capturing a representative sample including underserved groups. Primary research and systematic reviews were included. Guidelines and/or toolkits were included if they were applicable to the health research discipline without modification. Studies were excluded if they presented existing (unchanged) guidelines and/or toolkits previously identified elsewhere, did not explicitly describe a comprehensive framework and/or toolkit but rather reported on a single study area (for example, qualitative research with a specific underserved group), were published as abstracts only, identified underrepresented groups in research and/or emphasized the need for further research relating to underrepresented groups, or described frameworks and guidelines intended to support the engagement and retention of underrepresented groups in research but not the process of ascertaining who should be included in the sample.

### Data extraction, synthesis and draft toolkit development

Data were extracted from documents meeting the eligibility criteria using a bespoke predesigned form in Microsoft Excel. Quality appraisal and risk-of-bias assessment were planned but not possible due to the unavailability of relevant appraisal tools. Two researchers (A.R., F.K.) independently extracted data on document authorship, origin, date and characteristics of the methods described in the document, including target population and components of the methods used. A framework^78^ was developed according to the stages of research proposal development (namely, conceptualizing aims, methods, analysis and reporting), forming the basis of the draft REP-EQUITY toolkit. The extracted systematic review data were categorized into the stages of the framework, formulated into a series of methodological steps and descriptively presented (Supplementary Fig. 2). The draft toolkit was shared during the consensus workshop, and its contents and presentation were iteratively amended based on participant feedback.

### Consensus workshop sample selection and recruitment

Purposive sampling was used to recruit participants. The NIHR Birmingham BRC convened an equity, diversity and inclusion PPIE panel and senior academics in leadership roles across the breadth of research undertaken in Birmingham BRC (namely, early-phase, translational research in inflammatory arthritis; sarcopenia and multimorbidity; inflammatory liver disease; cancer inflammation; patient-reported outcomes; data, diagnostics and decision tools; infection and acute care; metabolic health in women; thrombo-inflammation; oral, intestinal and systemic health; and next-generation therapies). Approaches were made by email by the BRC operations team and the public involvement advisor overseeing public contribution. Recruitment continued on a rolling basis until each research theme was represented. Participants were not recruited on the basis of their personal characteristics, such as gender identity or ethnicity, and these data were not collected for workshop participants. The sample was determined through estimations of feasibility based on the experience of carrying out similar hybrid workshops to maximize interaction and data generation^79,80^.

Individuals indicating interest in participating in the workshop were provided with a participant information sheet and consent form. Accessibility was promoted by offering participants a range of consenting options (electronic Word document, online Microsoft form, verbal consent by telephone) and the opportunity to discuss the research before providing consent. Consent was obtained before the workshop. Public contributor participants were offered honoraria for their time, and expenses incurred during the workshop and the preparation session were recompensated.

### Data collection during the consensus workshop

Workshop discussions were led by a female researcher with postdoctoral experience in qualitative methodology (A.R.), and a female qualitative doctoral researcher served as a scribe (B.C.); both of them were assisted by a female research associate (F.K.). One participant was well known to the lead researcher, whereas others had limited or no prior relationship to the research team except by what was disclosed in the recruitment material and available in the public domain. Only the research team and participants were present during the workshop. The research team members were not blinded to the study aims and hypotheses. Attendance of the workshop was offered in person at the University of Birmingham and remotely through Zoom videoconferencing software (version 5.16). The workshop consisted of a series of presentations and facilitated discussions to draw upon the group’s expertise. Discussions were conducted using a semistructured topic guide consisting of open-ended questions to allow exploration of novel topics (Supplementary Appendix 1). Data collection was not informed by data saturation^81^ due to the one-off, time-limited nature of the workshop. Thresholds for consensus were planned whereby decisions proceeded if ratified by ≥70% of the group. In the case of <100% consensus, decisions were discussed until those in disagreement were satisfied that their views had been considered.

Discussion topics included the utility of the proposed toolkit, its applicability to the participants’ work, any gaps or required changes, and perceived potential barriers and facilitators to its use. Participants were divided into three groups (two in-person groups and one remote group), and discussions were facilitated by a team member, after which discussions were shared with the main group. Participants were divided for the focus group discussions pragmatically whereby remote participants were placed together in a discussion group. In-person participants were each placed alternately in groups 1 and 2 in the order in which they were seated. The workshop was recorded using encrypted equipment, and detailed notes, including direct quotes, were taken describing discussions and observations of how the participants interpreted the REP-EQUITY toolkit. Discussions were summarized, and the recordings were used for verification. After the meeting, the presentations and draft REP-EQUITY toolkit were reshared with those who wished to contribute further. Public contributor participants shared feedback on their participation experience following the workshop.

### Qualitative analysis and synthesis

Based on the participants’ comments and the research team’s observations of how the participants interpreted the toolkit contents, the research team iteratively amended and further refined the presentation and wording of the toolkit by developing a retrospective case study.

A deductive rapid analysis approach was used^82^ whereby, immediately after the workshop, the scribe reviewed the notes, indicating when additional information or a timestamp was needed. The lead researcher then reviewed and edited the notes while listening to the audio recordings, building upon the scribe’s notes. After reading the notes, the research team developed an initial coding framework based on the workshop aims to explore the utility of the proposed REP-EQUITY toolkit and the barriers and facilitators to its use. Additional codes were developed and included as the analysis progressed, and the framework was modified accordingly to ensure that new themes were captured^83^. The coding frame and sample codes were generated independently by the lead researcher and discussed within the research team. Disagreements were discussed until resolved. Proposals for implementation and eventual use were formulated.

### Statistical analysis

Statistical analyses were not performed in this study.

### Reporting summary

Further information on research design is available in the Nature Portfolio Reporting Summary linked to this article.

## Online content

Any methods, additional references, Nature Portfolio reporting summaries, source data, extended data, supplementary information, acknowledgements, peer review information; details of author contributions and competing interests; and statements of data and code availability are available at 10.1038/s41591-023-02665-1.

### Supplementary information

Supplementary Information Supplementary Figs. 1 and 2, Tables 1–4 and Appendixes 1 and 2.

Reporting Summary

## Data Availability

Embase, MEDLINE, Google (Google Search, Google Images and Google Scholar), Trip, National Grey Literature Collection and BASE databases were searched. Study data will be retained and securely stored by the University of Birmingham for 10 years from collection, after which time they will be securely destroyed. The unedited workshop recordings contain identifiable data and cannot be shared. The anonymized detailed workshop summaries are available in Supplementary Appendix 2.
