# Supplementary material for: A toolkit for capturing a representative and equitable sample in health research
Source: Nat Med. 2023 Dec 8;29(12):3259–67. doi: 10.1038/s41591-023-02665-1 (PMC10719102; doi:10.1038/s41591-023-02665-1)
Supplement: Supplementary file 1 — Supplementary Figs. 1 and 2, Tables 1–4 and Appendixes 1 and 2. [file 41591_2023_2665_MOESM1_ESM.pdf]

nature medicine

Analysis https://doi.org/10.1038/s41591-023-02665-1

A toolkit for capturing a representative and equitable sample in health research

In the format provided by the authors and unedited

Supplementary Figure 1: PRISMA diagram

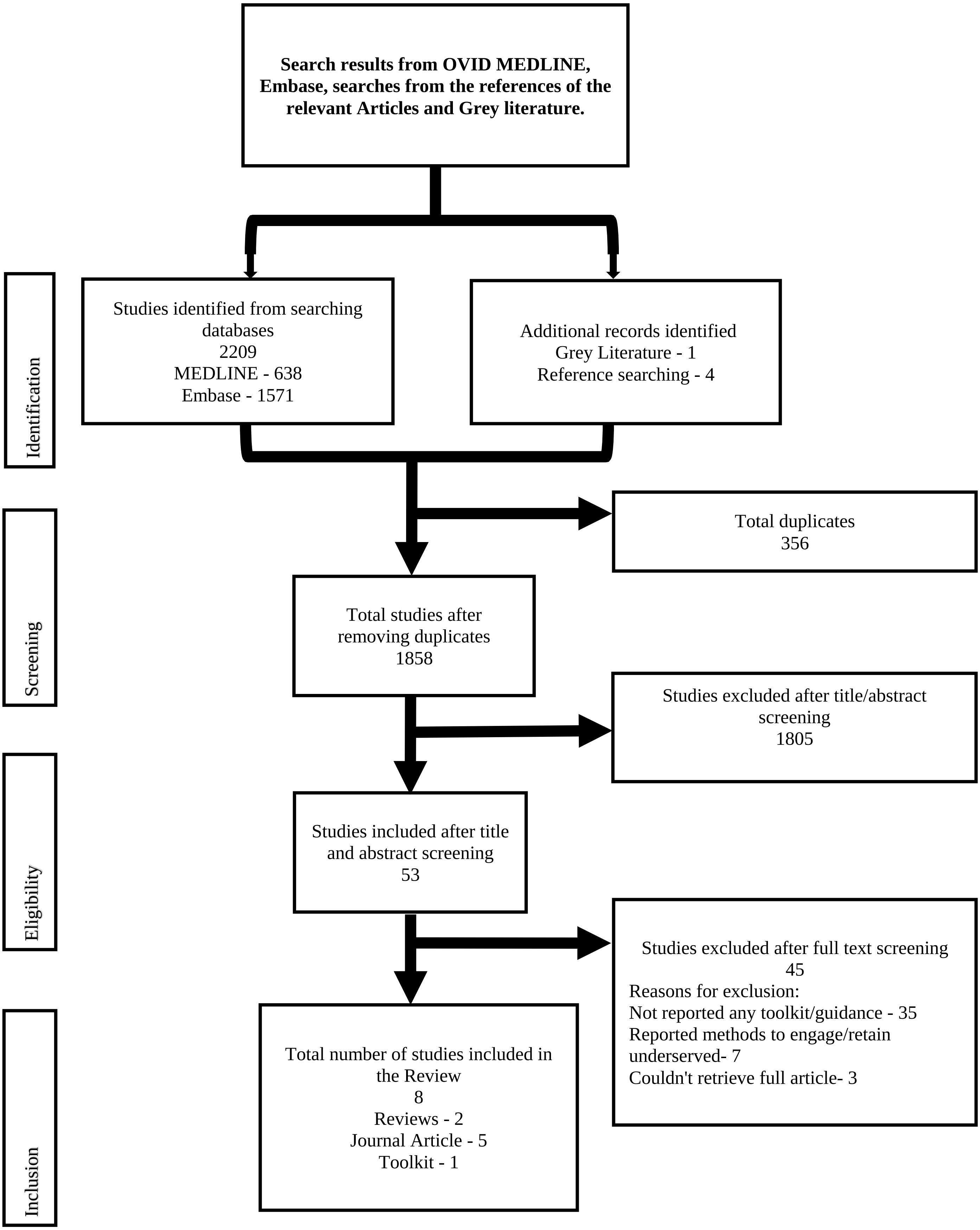

Supplementary Table 1: Characteristics of the included documents

Author, Year, Country	Target population	Aims	Methodology					Reporting framework or methods
			Literature review / systematic review	Stakeholder Involvement/ Engagement	Stakeholder Input	Consensus methods	Use of population-level data	
Cespedes 2022, USA	Black, Hispanic/ Latinx, cisgender men who have sex with men, transgender women, transgender men, and non-binary populations	Describe strategic approaches undertaken in the PURPOSE 2 trial to optimise engagement of underrepresented individuals.	Literature review	Engaged with community and patient stakeholders in US and globally. Formation of Global Community Advisory and Accountability Group (GCAG).	GCAG provided ongoing input on community needs, advised study team, site investigators, and staff. Collaboratively developed criteria for site selection, participant eligibility and demographic inclusion goals.	Global community forums, roundtable discussions, and individual meetings.	Reported site selection based on high rates of new HIV infection, disproportionate impact/burden of HIV, historical HIV incidence data.	Methods
Bolen 2005, USA	Racial and ethnic minorities, older adults, adolescents, rural populations, and individuals of low socio-economic status	1) Explore challenges and strengths in choosing an a priori definition of recruitment success and available measurement approaches. 2) Formulate a framework for choosing consistent a priori recruitment goals for underrepresented groups based on the research question and study location.	Systematic review	Not reported	Not reported	Not reported	Advises investigators to adjust for disease prevalence or mortality when using geographic proportions of underrepresented groups.	Framework

Pratt 2021, Australia	Marginalised or vulnerable groups in high-income countries and low- and middle-income countries	Describe framework to ethically guide global health researchers and funders on how to design research projects and grants programmes to promote global health equity.	Literature review	Researchers, international community partners, ethics advisory board members, trial participants, research funder portfolio advisors.	Participation in in-depth interviews, direct observation, priority-setting exercises, case-studies.	Not reported	Advises use of data, including health status and multidimensional poverty metrics, to inform population selection.	Framework
O'Reilly-de Brún 2016, Republic of Ireland	Undocumented migrants, refugees, people seeking protection (asylum seekers) and low-income economic migrants	Describe Participatory Learning and Action methodology, mode of engagement and techniques used to enhance migrants' access and engagement in research.	Not reported	Community interpreters trained as peer researchers	Co-generated a sampling frame and co-designed and translated recruitment leaflets	Charting, ranking, mapping and assessment techniques, interviews and focus groups.	Not reported	Methods
Godden 2010, UK	Minority ethnic groups	Explore the feasibility of designing a study to evaluate representation of minority ethnic groups in clinical cancer research trials.	Not reported	Not reported	Not reported	Not reported	Reported using a range of routine data to profile representation by ethnicity	Methods

Dowling 2012, USA	Older adults including those of low-income, minority, and rural populations.	Describe design of participant recruitment registry for clinical studies of older adults.	Not reported	Clinicians, study coordinators, administrators, information technologists.	Identify recruitment processes and strategy.	Consensus meetings to reach agreement between stakeholders and development team.	Reported systematic collection of anonymous, de-identified demographic data to gauge recruitment effectiveness and evaluate registry population.	Methods
Farooqi 2018, UK	Black, Asian and Minority Ethnic (BAME) groups	Describe best practice and framework on how to improve participation of Black, Asian and Minority Ethnic groups in research.	Literature Review	Researchers, members of the public belonging to BAME community.	Exploration of enablers and barriers to research conduct and formulation of good practice guidelines.	Workshops.	Advises use of prevalence and primary care data.	Framework
Corbie-Smith 2004, USA	Women and minorities	1) Describe interpretations of National Institute for Health guidance.<br>2) Describe framework for considering the inclusion of minorities in research.	Not reported	Not reported	Not reported	Not reported	Advises use of data to ascertain disease burden by group, and guide sample proportions.	Framework

Supplementary Table 2: Systematic literature search strategy, Ovid

Ovid MEDLINE(R) <1946 to September Week 1 2022>

1	Minority Groups/
2	underrepresented.mp.
3	hard to reach.mp.
4	difficult to reach.mp.
5	disadvantaged.mp.
6	deprived.mp.
7	hidden population.mp.
8	invisible population.mp.
9	marginali*.mp.
10	1 or 2 or 3 or 4 or 5 or 6 or 7 or 8 or 9
11	Research Design/
12	Research/
13	Health Services Research/
14	Clinical Trial/
15	clinical research.mp.
16	11 or 12 or 13 or 14 or 15
17	framework*.ti,ab.
18	guideline*.ti,ab.
19	tool*.ti,ab.
20	checklist*.ti,ab.
21	standard*.ti,ab.
22	17 or 18 or 19 or 20 or 21
23	equity.ti,ab.
24	equality.ti,ab.
25	divers*.ti,ab.
26	inclu*.ti,ab.
27	23 or 24 or 25 or 26
28	10 or 16 or 22 or 27
29	10 and 16 and 22
30	10 and 16 and 22 and 27

Supplementary Table 3: Systematic literature search strategy, Embase

Embase <1974 to 2022 October 10>

1	Minority Groups.mp. or minority group/
2	underrepresented.mp.
3	hard to reach.mp.
4	difficult to reach.mp.
5	disadvantaged.mp.
6	deprived.mp.
7	hidden population.mp.
8	invisible population.mp.
9	marginali*.mp.
10	1 or 2 or 3 or 4 or 5 or 6 or 7 or 8 or 9
11	Research Design/
12	Research/
13	Health Services Research/
14	Clinical Trial/
15	clinical research.mp. or clinical research/
16	11 or 12 or 13 or 14 or 15
17	framework*.ti,ab.
18	guideline*.ti,ab.
19	tool*.ti,ab.
20	checklist*.ti,ab.
21	standard*.ti,ab.
22	17 or 18 or 19 or 20 or 21
23	equity.ti,ab.
24	equality.ti,ab.
25	divers*.ti,ab.
26	inclu*.ti,ab.
27	23 or 24 or 25 or 26
28	10 and 16 and 22
29	10 and 16 and 22 and 27

Supplementary Table 4: Summarised grey literature search strategy

1	Representative sample in medical research of underrepresented groups
2	Representative sample in medical research of minority groups
3	Representative sample in medical research of minorities
4	Representative sample in medical research of underserved groups
5	Representative sample inclusion of minorities
6	Underserved groups
7	Representative sample
8	Minority research
9	Representative research sample
10	Research framework
11	Representative
12	Underserved Bame in clinical research
13	Underserved Black-African in clinical research
14	Underserved Asian in clinical research
15	Underserved Chinese in clinical research
16	Underserved Indian in clinical research
17	Underserved Pakistani in clinical research
18	Underserved Bangladeshi in clinical research
19	Underserved Arab in clinical research
20	Underserved Youth in clinical research
21	Underserved Elderly in clinical research
22	Underserved LGBTQ in clinical research
23	Underserved Gender (identity) in clinical research

Supplementary Figure 2: Draft Toolkit

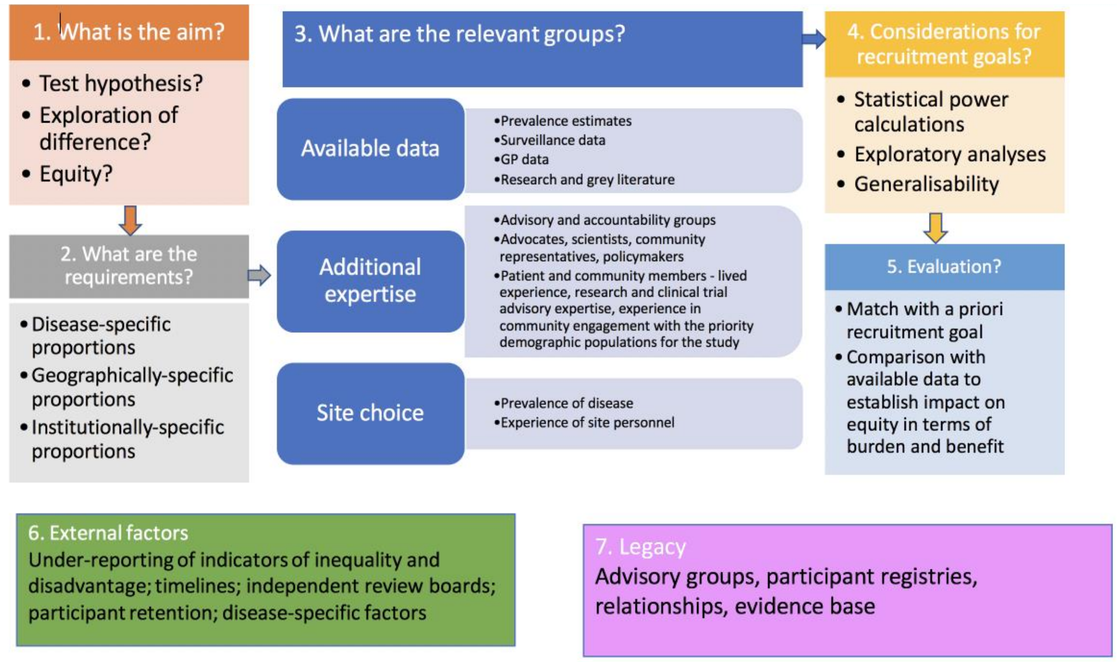

Supplementary Appendix 1: Workshop Topic Guide

This is the preliminary topic guide. The overarching objectives will remain the same, but questions and prompts will be developed as the discussion takes place to incorporate any important themes that emerge.
TOPICS TO BE COVERED IN THE MEETING
Discussion points will centre on draft toolkit, exploring:

(1) Initial views of draft Toolkit
- Are there any gaps?
- Does anything not make sense?
- What do you think of the steps

(2) Acceptability of proposed Toolkit
- How do you feel about using such a Toolkit?
- How does it align with your research priorities?

(3) Feasibility of their implementation
- Do you think the Toolkit makes sense in your research?
- How do you think its use would fit?
- Are there any reasons you would not use the Toolkit?
- What support do you think would be needed?

Supplementary Appendix 2: Workshop Summary Notes

1. Is the Toolkit useful?

- How would it work for rare diseases?
- Timing: When should I use this guidance – does this apply for trials with small sample size?
- What does the evidence tell us? Which factors are a driving response?
- The guidance is needed but important to not end up stating “We tried but did not achieve it.”
- When using patient-derived samples (pre-clinical but going more into translational space) - when thinking about the population of those patient that we are analysing and proportion of ethnic minorities and gender diversity etc., there are things within this toolkit that allows seeing them all in one place, which is quite helpful – “a bit of a lightbulb moment, really”
- Working in fundamental science, this is quite removed
- Being exposed to a lot of this but because not being involved in designing or leading a trial makes it difficult to respond to the presented design
- Doing experiments ensuring a range of diversity is difficult

- Teams have started to match samples we recruited to the population using the local hospital's health records; but it is still difficult to ensure you are being fully inclusive and that you have got a diverse data set when dealing with small sample size

- Conducting research using a national survey to look at clinical population versus the general population. But when looking at research papers that have the same biases as we have, you may be comparing good practices to good practices, or bad to bad?
- Participant numbers as requirement for conducting your research and risk of trial closure: falling short with 3 to 4 people and not being able to conduct/complete the study
  - Recruiting patient through hospitals to ensure reaching certain number of participants
- Recruitment/Source of patients/patient data is crucial for the representativeness of any conducted research using this toolkit e.g., having multiple sites of recruitment (Locations)
  - More sites, more diversity? Depends on the area where we link patient representation to e.g., areas with small ethnic minority populations – Hence, yes, more sites benefit but also disadvantages. Even hyper-diverse cities are difficult to rely on for recruitment of large trials.
- Implementing the tool depends on funder's interest in investing in your research
  - E.g., Certain funders do not express interest in this
    - Look to example of patient and public involvement and engagement (PPIE) where originally, it was 'nice to do' and not 'necessary to do' as there was push-back from certain funders and additional PPIE costs made grant applications uncompetitive.
  - For research centre in question: resource implication and being able to access the resource quite quickly would be a challenge that the centre would need to support/facilitate
  - Thus, funders need to be aware and supportive of the implementation of this toolkit as, if they are not, researchers cannot implement this.
  - It can become a disadvantage for your application when applying for grants explaining extra costs for the implementation of the toolkit.
- Considerable interest among statisticians in diversity and including underserved groups, but they wanted advice on 'how to work it out' and 'where to go to progress it'.

2. What are the barriers for use of the Toolkit?

- Consider how comfortable people are with sharing their data. People may be used to being asked about things like ethnicity but in cases where data relating to gender identity or marital status, for example, are requested, people may be wondering how these data will be used. There is a trend whereby people are asked to share information on the nine protected characteristics in the UK Equality Act 2010, but onus on researcher to rationalise what is "nice to know versus need to know"
- Researchers must earn the public's trust in relation to sharing their data.
- Trial teams under pressure to recruit in a set time-frame or close, and a time-consuming sampling and recruitment process may not be compatible.
- For basic fundamental science - how do you get access to the required information?
- Expectation of any given study: In the beginning, you expect pragmatic evaluation of it but then in the context, that may not be possible due to the number of participants. So, you set your aim, but you may not achieve.
- Concern: can we go out and engage with community groups, them get to wanting to engage in research and then look at factors for representation?
- What is the upfront time, commitment, and resource implication to do this?
  - access to resources to assist with this is probably needed
  - A reflection on where our own individual research sits on the grand scheme of things, and therefore, where are we starting in this process? What are the disease-specific proportions and looking at it from that point of view.
- A lot of this will be really difficult for people that do not have access to patients and do not have access to data about patient groups or demographics. It is much easier, probably, if you are in applied health research or data research. But if you're doing basic or fundamental science, how would you get access to this information? Other than looking at other studies, which could just have the same biases from research that has been done without this guidance.

    - Research centres could utilise existing health data and flag this material for researchers, because for some this is just inaccessible.
- Ethnicity and the challenges, especially on the genomics work, so we just expect pragmatic evaluation of that. Now we discussed the deprivation, and how you might assess that, and you can go down that long list. But then, in the context of a ten, to fifteen to twenty-five patients' study that may not be possible to go all the way down the list. I think we just require something pragmatic, that gives you a sense of "if I am doing that study in this region; This is broadly what I expect to look like, and then how you achieve that".
    - We might not be able to achieve that, but at least you would be clear as to what it should look like, and how you might do that, and if it does not work the next time around, you can say, well, what do we need to do to improve that going forward. That's still a lot further forward than the way we currently undertake studies.

3. What are the facilitators for its use?

- Design of studies contributes to the engagement of people
    - The default should be if you design a study to do it in a pragmatic way.
    - Do not just pick a certain number of certain characteristics but consider gaps in representation in the respective research area
- Having case studies describing different situations will be helpful
    - While it is obvious that EDI is well established in clinical research, other researchers such as those engaged in health data and methodologies may not find immediate relevance. It is for them to ask the question: would you be intersecting with translational research? Will your specialised field have knowledge or health relevance in the foreseeable future to improve human health, or would the effect be aligned with public trust and confidence and therefore impinge on the sustainability of your discipline from the public support perspective? Sometimes at a minimal level, you might have a duty to share your knowledge to inform, be publicly accountable, and to empower the public. In support of this, case studies could be made widely available to exemplify how good EDI can be done. They could include how underserved communities could be involved and engaged.
        - There could be more support provided to fill gaps in understanding, in the form of more knowledge sharing through training, discussion forums between disciplines and involving people with insights and experience in EDI in research. It therefore seems imperative that a case will need to be made by each researcher in the first instance. If it is strong enough, and patient and public benefit can be accounted for, then it stands a much better chance of getting funded.
