# Supplementary material for: A toolkit for capturing a representative and equitable sample in health research
Source: Nat Med. 2023 Dec 8;29(12):3259–67. doi: 10.1038/s41591-023-02665-1 (PMC10719102; doi:10.1038/s41591-023-02665-1)
Supplement: Supplementary file 2 — Reporting Summary [file 41591_2023_2665_MOESM2_ESM.pdf]

nature portfolio

Corresponding author(s): Ameeta Retzer

Last updated by author(s): Oct 23, 2023

Reporting Summary

Nature Portfolio wishes to improve the reproducibility of the work that we publish. This form provides structure for consistency and transparency in reporting. For further information on Nature Portfolio policies, see our Editorial Policies and the Editorial Policy Checklist.

Statistics

For all statistical analyses, confirm that the following items are present in the figure legend, table legend, main text, or Methods section.

n/a	Confirmed	
☒	☐	The exact sample size (*n*) for each experimental group/condition, given as a discrete number and unit of measurement
☒	☐	A statement on whether measurements were taken from distinct samples or whether the same sample was measured repeatedly
☒	☐	The statistical test(s) used AND whether they are one- or two-sided<br>*Only common tests should be described solely by name; describe more complex techniques in the Methods section.*
☒	☐	A description of all covariates tested
☒	☐	A description of any assumptions or corrections, such as tests of normality and adjustment for multiple comparisons
☒	☐	A full description of the statistical parameters including central tendency (e.g. means) or other basic estimates (e.g. regression coefficient) AND variation (e.g. standard deviation) or associated estimates of uncertainty (e.g. confidence intervals)
☒	☐	For null hypothesis testing, the test statistic (e.g. *F*, *t*, *r*) with confidence intervals, effect sizes, degrees of freedom and *P* value noted<br>*Give P values as exact values whenever suitable.*
☒	☐	For Bayesian analysis, information on the choice of priors and Markov chain Monte Carlo settings
☒	☐	For hierarchical and complex designs, identification of the appropriate level for tests and full reporting of outcomes
☒	☐	Estimates of effect sizes (e.g. Cohen's *d*, Pearson's *r*), indicating how they were calculated

Our web collection on statistics for biologists contains articles on many of the points above.

Software and code

Policy information about availability of computer code

Data collection	Endnote (Version X9), Microsoft Excel and Word (Version 16.43, 2020), Microsoft Forms, Zoom (Version 5.16)
Data analysis	Microsoft Excel (Version 16.43, 2020)

For manuscripts utilizing custom algorithms or software that are central to the research but not yet described in published literature, software must be made available to editors and reviewers. We strongly encourage code deposition in a community repository (e.g. GitHub). See the Nature Portfolio guidelines for submitting code & software for further information.

Data

Policy information about availability of data

All manuscripts must include a data availability statement. This statement should provide the following information, where applicable:

- Accession codes, unique identifiers, or web links for publicly available datasets
- A description of any restrictions on data availability
- For clinical datasets or third party data, please ensure that the statement adheres to our policy

Embase, MEDLINE, Google (Google search, Google images and Google scholar), Trip, National Grey Literature Collection and BASE databases were searched. Study data will be retained and securely stored by the University of Birmingham for 10 years post-collection, after which time it will be securely destroyed. The unedited workshop recordings contain identifiable data and cannot be shared. The anonymised detailed workshop summaries are available in Supplementary Appendix 2.

Research involving human participants, their data, or biological material

Policy information about studies with human participants or human data. See also policy information about sex, gender (identity/presentation), and sexual orientation and race, ethnicity and racism.

Reporting on sex and gender	These data have not been collected.
Reporting on race, ethnicity, or other socially relevant groupings	These data have not been collected.
Population characteristics	See above.
Recruitment	Individuals were recruited form the National Institute for Health and Care Research (NIHR) Birmingham Biomedical Research Centre (BRC) senior research team and the NIHR Birmingham BRC Equity, Diversity and Inclusion (EDI) public and patient involvement panel. Approaches were via email by the BRC operations team and the public involvement advisor overseeing public contribution. Recruitment continued on a rolling basis until each research theme was represented. Individuals indicating interest in participating in the workshop were provided with a participant information sheet and consent form. To promote accessibility, participants were offered a range of consenting options (electronic Word document, online Microsoft form, verbal consent via telephone) and the opportunity to discuss the research in advance of giving consent. Consent was taken prior to the workshop. Public contributor participants were remunerated for their time and expenses incurred during the workshop and preparation session. Self-selection bias was possible as participants were those likely to be interested in the topic, however, recruitment material did not indicated experience of EDI methodology was at all necessary but stated recruitment was on the basis of their own research experience. Participants were not recruited on the basis of personal characteristics, such as gender identity or ethnicity, and these data were not collected for workshop participants.
Ethics oversight	This study has undergone ethical review by the Research Ethics Committee at the University of Birmingham and was granted full approval in October 2022 (ERN_22-1182).

Note that full information on the approval of the study protocol must also be provided in the manuscript.

Field-specific reporting

Please select the one below that is the best fit for your research. If you are not sure, read the appropriate sections before making your selection.

☐ Life sciences ☒ Behavioural & social sciences ☐ Ecological, evolutionary & environmental sciences

For a reference copy of the document with all sections, see nature.com/documents/nr-reporting-summary-flat.pdf

Behavioural & social sciences study design

All studies must disclose on these points even when the disclosure is negative.

Study description	This was a mixed methods study involving a methodological systematic review and synthesis and a qualitative consensus workshop.
Research sample	Participants were: (1) senior researchers from across the breadth of research undertaken in the Birmingham BRC, namely early phase, translational research in inflammatory arthritis, sarcopenia and multimorbidity; inflammatory liver disease; cancer inflammation; patient-reported outcomes; data, diagnostics and decision tools; infection and acute care; metabolic health in women; thrombo-inflammation; oral, intestinal and systemic health; and next generation therapies; and (2) patient and members of the public that formed the NIHR Birmingham EDI public and patient advisory panel. Demographic information was not collected but participants were recruited on the basis of their experience leading research and/or providing public contribution. This pool of participants was chosen because the research undertaken in the Birmingham BRC is representative of a broad range of research areas, impacting a diverse range of individuals and patient groups, and all have experience of research relating to populations residing in Birmingham, UK, a super-diverse city. However, the participants' experience relates to early phase and translational research and as such, their insights may not represent those in other areas of health research such as applied health research and data science. However, members of the research team are experienced in applied health research, mediating this to a degree. Participants were not recruited on the basis of their personal characteristics, such as gender identity or ethnicity, and these data were not collected for workshop participants.<br>To identify academic and grey literature, Embase and MEDLINE, Google (Google search, Google images and Google scholar), Trip database, National Grey Literature Collection database and BASE databases were searched. Pertinent articles may have been missed in the literature search due to the decision to restrict to two academic databases. While this was mitigated through backward and forward citation searching; extensive grey literature searches; and developing strategies from previous systematic reviews, non-English language literature was excluded and though several grey literature databases were used, searches via Google would be geo-tagged and linked with UK sources, so unrepresentative of international non-academic literature.
Sampling strategy	Purposive sampling was used to recruit workshop participants. The workshop was a one-off qualitative exercise with a fixed period of time, so seeking data saturation was not possible in this case. The sample was determined through estimations of feasibility based on experience of carrying out similar hybrid workshops to maximise interaction and data generation (doi: 10.1093/noajnl/vdad096, doi: 10.1371/journal.pone.0240518). Participants were given the opportunity to contribute further through review of workshop summaries and the study team's interpretation of their data.

	Literature databases were searched without date limitation. The search strategy included Medical Subject Headings (MeSH) terms, broad search terms, phrases and keywords related to equity, diversity and inclusion in health research, accounting for variation in terminology used in this area (Supplementary Tables 2 and 3). These were refined through scoping searches, informed by strategies used in comparable systematic reviews. Forward and backward citation searching were used. Search terms and keywords were derived from the academic literature review search strategy and refined iteratively in consultation with search specialists and the patient and public involvement and engagement panel. Search terms were piloted in each database in a process where the retrieved records were checked for sensitivity and the terms re-worked and re-entered items that related to our aim were retrieved (Supplementary Table 4). Upon advice from the patient and public involvement panel, new terms were introduced based on those commonly used in the United Kingdom relating to ethnicity, reflecting categories used in census data.
Data collection	The workshop consisted of a series of presentations and facilitated discussions to draw upon the expertise of the group. Discussion topics related to the utility of the proposed Toolkit and any gaps or required changes, and potential barriers and facilitators for its use. The workshop was recorded using encrypted equipment and notes were taken. Discussions were summarised and the recordings used for verification. The presentations and draft Toolkit were re-shared with participants following the meeting for those who wished to contribute further. Those present were only the research team and participants. The research team were not blinded to the study aims and hypotheses.
Timing	The two-hour hybrid format workshop took place on November 14 2022.
Data exclusions	No data were excluded from analyses.
Non-participation	Two individuals declined participation due to diary conflicts, two individuals intending to participate did not do so due to unanticipated diary conflicts.
Randomization	Randomization was not used for participant sampling for the consensus workshop, which was purposive. Participants were divided for the focus group discussions pragmatically, whereby those joining remotely were placed in a discussion together. Participants joining the workshop in person were each placed alternatively by the research team in groups 1 and 2 in the order in which they were seated.

Reporting for specific materials, systems and methods

We require information from authors about some types of materials, experimental systems and methods used in many studies. Here, indicate whether each material, system or method listed is relevant to your study. If you are not sure if a list item applies to your research, read the appropriate section before selecting a response.

Materials & experimental systems

n/a	Involved in the study
☒	☐ Antibodies
☒	☐ Eukaryotic cell lines
☒	☐ Palaeontology and archaeology
☒	☐ Animals and other organisms
☒	☐ Clinical data
☒	☐ Dual use research of concern
☒	☐ Plants

Methods

n/a	Involved in the study
☒	☐ ChIP-seq
☒	☐ Flow cytometry
☒	☐ MRI-based neuroimaging

Plants

Seed stocks	*Report on the source of all seed stocks or other plant material used. If applicable, state the seed stock centre and catalogue number. If plant specimens were collected from the field, describe the collection location, date and sampling procedures.*
Novel plant genotypes	*Describe the methods by which all novel plant genotypes were produced. This includes those generated by transgenic approaches, gene editing, chemical/radiation-based mutagenesis and hybridization. For transgenic lines, describe the transformation method, the number of independent lines analyzed and the generation upon which experiments were performed. For gene-edited lines, describe the editor used, the endogenous sequence targeted for editing, the targeting guide RNA sequence (if applicable) and how the editor was applied.*
Authentication	*Describe any authentication procedures for each seed stock used or novel genotype generated. Describe any experiments used to assess the effect of a mutation and, where applicable, how potential secondary effects (e.g. second site T-DNA insertions, mosiacism, off-target gene editing) were examined.*
